# The Neuropathology of Alcohol Use Disorder: Cellular Insights From Human Post‐Mortem Studies

**DOI:** 10.1111/jnc.70233

**Published:** 2025-09-11

**Authors:** Ameer E. Rasool, Jennifer L. Cornish, Asheeta A. Prasad

**Affiliations:** ^1^ School of Medical Sciences, Faculty of Medicine and Health University of Sydney Sydney New South Wales Australia; ^2^ School of Psychological Sciences, Faculty of Medicine Health and Human Sciences, Macquarie University Sydney New South Wales Australia

**Keywords:** alcohol use disorder, neuropathology, post‐mortem human brain

## Abstract

Alcohol use disorder (AUD) is a complex neurological disorder with limited treatments available. Thus, understanding the neurobiological changes associated with AUD is crucial for the development of effective therapeutic approaches. Analysis of human post‐mortem tissue provides insight into the long‐term effects of alcohol use and cellular changes that contribute to addiction behavior. Here, we provide a collection of cellular changes found in human AUD post‐mortem brain tissue, revealing brain region‐specific cellular adaptations that map a complex neurobiological landscape of addiction. Examination across the cortex, striatum, hippocampus, hypothalamus, cerebellum, and midbrain reveals that although degeneration, metabolic disruption, and neuroinflammatory processes are common cellular processes that are impacted, these changes are distinctive by brain region and cell type. For example, white matter loss dominates in the prefrontal cortex, the hippocampus is sensitive to glial cell loss, subtypes of hypothalamic neurons are disproportionately affected, and the striatum shows subregional changes. Although these cellular adaptations are brain region and cell type specific, this review of studies over the last 30 years suggests that neuropathology in AUD undergoes neural network reorganization. Whether these changes are a response to chronic alcohol use or underlying alcohol‐seeking behavior is a limitation of post‐mortem analysis. However, mechanistic studies of rodent models and convergent evidence from human post‐mortem tissue have identified therapeutic candidates such as oxytocin and GLP‐1. Insights from animal studies have also highlighted underexplored yet potentially pivotal regions in AUD, such as the anterior insular cortex and the ventral pallidum, in human studies. Other limitations include a lack of sex‐specific analyses, the incorporation of advanced neuroscience tools, and multiple regional analyses. Integrating AUD human post‐mortem tissue bridges preclinical and clinical research, providing an invaluable understanding of neural mechanisms underpinning AUD and potential avenues for targeted interventions.

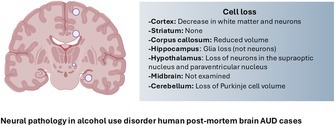

## Graphical abstract

Major cellular changes in alcohol use disorder human post‐mortem brain. Alcohol use disorder (AUD) is a complex neurological disorder that undergoes neural changes, mainly degeneration and or cellular alterations impacting addiction behaviours. Key brain regions implicated in AUD are the cortex, striatum, corpus callosum, hippocampus, hypothalamus, midbrain and cerebellum. This review provides an overview of the key brain regions that undergo cell loss and cellular plasticity using post mortem human brains cases from AUD cases.

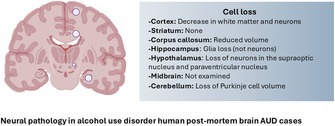

**Abbreviations**
AUD: alcohol use disorder
BA9: brodmann area 9
dlPFC: dorsolateral PFC
fMRI: functional magnetic resonance imaging
GLP‐1: glucagon‐like peptide‐1
GLP‐1R: glucagon‐like peptide‐1 receptor
IFNγ: interferon gamma
LC–MS/MS: liquid chromatography–tandem mass spectrometry
M4: muscarinic acetylcholine receptor (mAChR)
MOR: μ‐opioid receptor
mRNA: messenger ribonucleic acid
NAc: nucleus accumbens
OC: occipital cortex
PET: positron emission tomography
Peth: phospholipase D produces phosphatidyl ethanol
PFC: prefrontal cortex
PKR: protein kinase R
PVN: paraventricular nucleus
RIN: RNA integrity number
RNA: ribonucleic acid
SGF: superior frontal gyrus
SN: substantia nigra
SON: supraoptic nucleus
TLR: toll‐like receptor
VTA: ventral tegmental area

## 1 Introduction

Alcohol use disorder (AUD) is characterised by cycles of abuse, abstinence, withdrawal, and relapse (Everitt et al. 2001). The fifth edition of the Diagnostic and Statistical Manual of Mental Disorders (DSM–5) revised AUD into a single disorder, integrating alcohol abuse and alcohol dependence. The rate of AUD is increasing globally (Glantz et al. 2020; Verplaetse et al. 2021). However, there is a high prevalence of AUD, and therapies available for treatment remain limited and lack efficacy. Therefore, further understanding of the pathology of AUD is essential in developing effective treatments. Approaches to better understand the neurobiology and pathology associated with AUD include pre‐clinical models of AUD and cellular investigation analyses in post‐mortem human brain sections. While pre‐clinical animal studies allow behavioral and neural manipulation with deeper insight into cellular mechanisms, there are translational limitations. A significant strength of post‐mortem human brain analyses is the detailed observation of the long‐term impact of AUD, which is challenging to identify in pre‐clinical models. Here, we attempt to compile the vast number of studies using post‐mortem human brains from subjects with AUD to provide a collection of the neurobiological changes in this disorder. The brain regions impacted in AUD share overlapping neural circuitry with reward processing pathways, and those associated with the three key stages of addiction: binge and intoxication, negative affect and withdrawal, and anticipation and craving (Yang et al. 2022). Thus, neurobiological changes in AUD are most pronounced in the cortex, striatum, and hippocampus (Dedova et al. 2009).

Examining human post‐mortem brain tissue through gross anatomical and volumetric analyses has enriched our comprehension of the neural consequences of AUD by revealing specific structural alterations in select brain regions. The overall brain weight is lower in AUD than controls, with atrophy predominantly located in the frontal lobes (Harper et al. 2003; Sutherland et al. 2014). This decrease in brain weight correlates with lifetime alcohol consumption and is primarily due to loss of white matter (Harper 1998; Kril et al. 1997). Specifically, brain volume analysis indicated that distinct regions were notably smaller in individuals with chronic AUD than in controls (Kril et al. 1997). White matter areas, such as in the frontal lobe, were most significantly reduced in volume in addition to the medial temporal lobe and thalamus (Kril et al. 1997). Moreover, the same study revealed a decrease of approximately 15%–23% of neurons in the frontal cortex (Kril et al. 1997). However, this decrease was not associated with a significant reduction in volume in this region, thus suggesting that a substantial amount of tissue degeneration is required for gross volume changes (Kril et al. 1997). For comparative gene expression changes in brain regions of human AUD cases, see (Flatscher‐Bader et al. 2006). While several reviews have reported anatomical and volumetric changes, the current review provides a detailed collection of cellular changes in human post‐mortem brain areas.

## 2 Prefrontal Cortex

The prefrontal cortex (PFC) plays an essential role in modulating addictive behaviors by regulating executive function, decision‐making, and behavioral control (Everitt and Robbins 2005; Kalivas and Volkow 2005). Chronic alcohol consumption has been linked to structural and functional changes in the PFC. For instance, Positron Emission Tomography (PET) and Functional Magnetic Resonance Imaging (fMRI) studies have found a reduced volume of gray and white matter in the PFC in AUD, which has been associated with impairment of cognitive function such as impaired judgment, increased impulsivity, and a reduced ability to resist drug‐seeking behaviors (Dao‐Castellana et al. 1998; Goldstein et al. 2004; Okvist et al. 2007; Sullivan and Pfefferbaum 2005). Moreover, histological analysis has shown that the density of neurons in the PFC is reduced in AUD (Harper et al. 2003). More recently, neurolipidomic analysis of the frontal lobe identified a significant decrease in the white matter of prefrontal, middle temporal, and visual cortices and has been linked to reduced expression of selective lipids: sphingolipids (sulfatides and ceramides) and phospholipids (de la Monte et al. 2018; Smith et al. 2022). These studies reveal that structural changes in PFC white matter are mainly due to reduced lipids.

### 2.1 Structural and Functional Proteins

Proteomic examinations of the PFC using human post‐mortem tissue offer insights into the mechanisms underlying the structural and functional changes associated with AUD. Using 2D gel electrophoresis analyses of protein expression in white matter in the dorsolateral PFC (dlPFC) Brodmann area 9 (BA9) in AUD, we found a general decline in protein expression (Alexander‐Kaufman et al. 2006). Specifically, metabolic enzymes essential for energy transduction, such as creatine kinase chain B, fructose‐biphosphate aldolase C, and glyceraldehyde‐3‐phosphate dehydrogenase (Alexander‐Kaufman et al. 2006) were reduced in AUD compared to healthy controls. Moreover, a decline in cytoplasmic protein hNP22 and cytoskeletal protein α‐internexin was also measured in white matter in dlPFC BA9 (Alexander‐Kaufman et al. 2006). Further examination of grey matter within the dlPFC demonstrated that although specific proteins exhibited comparable changes to those in white matter of the same region, there were also distinct trends (Alexander‐Kaufman et al. 2007). For instance, α‐internexin increased only in grey matter for individuals with AUD (Alexander‐Kaufman et al. 2007). Moreover, examination of hNP22 in the PFC using western blots revealed an increase in expression in the grey matter of BA9 in AUD, and immunohistochemical examination shows that hNP22 is restricted to the cytoplasm and processes of neurons and therefore may play a role in neuroplastic changes seen in AUD (Depaz et al. 2003). This suggests that while alcohol might influence specific cellular cascades, there could be varying mechanisms contributing to AUD‐related pathology in white and grey matter. These proteomic analyses of human post‐mortem PFC and validation using 2D gel electrophoresis, western blots, and immunohistochemistry have identified mechanisms behind the possible changes in cell metabolism that may underlie frontal lobe damage seen in AUD. Additionally, changes identified in cytoskeletal proteins may contribute to modifications in neuronal circuitry, enhancing susceptibility to addictive behaviors and AUD‐related brain damage.

### 2.2 Neurodegeneration

Proteins related to pro‐ and anti‐apoptotic events in human post mortem PFC were examined to determine a possible cause behind cell loss and neurodegeneration in AUD, and used western blotting to reveal pro‐apoptotic protein, caspase‐3, decreased, while anti‐apoptotic protein, B‐cell lymphoma‐2, and pro‐apoptotic caspase 8 increased (Johansson et al. 2009; Whittom et al. 2014). This finding was further supplemented by examining mRNA using qRT‐PCR, showing that pro‐apoptotic mRNAs were also downregulated (Johansson et al. 2009). These results from human post‐mortem tissue examination suggest that molecular adaptations in AUD may be aimed at mitigating the neurotoxic effects of chronic alcohol consumption and that cells with these protein and mRNA changes may possess the capability to reverse the effects of AUD in the PFC. Moreover, to further establish the molecular mechanisms behind brain tissue injury due to neurotoxicity induced by chronic alcohol consumption in the PFC, western blotting and immunofluorescence reveal changes in signal transduction pathways (Johnson et al. 2015). Specifically, interferon gamma (IFNγ), protein kinase R, and phosphorylated PKR, which have been implicated in neuronal apoptosis in other neurodegenerative diseases such as Alzheimer's disease, are increased in AUD subjects, thereby suggesting that activation of the IFNγ‐protein kinase R pathway may result in AUD‐related brain injury (Johnson et al. 2015).

Furthermore, toll‐like receptor (TLR) activation is observed in neurodegenerative diseases, including AUD; in particular, immunohistochemical examination of the human post‐mortem orbitofrontal cortex of subjects with AUD has shown an increase of TLR7 and tumor necrosis factor related apoptosis inducing ligand expression, which was accompanied by neuronal death (Qin et al. 2021). Additionally, within the human orbitofrontal cortex, there was upregulation of endoplasmic reticulum stress‐associated proteins, including glucose‐regulated protein 78, as well as oxidative stress markers, like 4‐hydroxynonenal (Qin et al. 2023). These increases were found using immunohistochemical methods alongside decreases in neuronal markers (NeuN and MAP2) and thus suggest another potential mechanism underlying AUD‐related cell death (Qin et al. 2023). In addition to neurons, astrocyte cell loss has been reported in the cortex and hippocampus. Within the cortex, the superior frontal cortex, when processed for TUNEL assay, an approach to detect DNA fragmentation as a measure of cell death found ~64% of the cases examined showed TUNEL‐positive co‐localization with GFAP immunoreactivity, indicating glial cells in the cortex are also under neurodegeneration (Ikegami et al. 2003).

### 2.3 Synaptic Plasticity

Proteomic analysis in the superior frontal gyrus (SFG) and the occipital cortex (OC) using 2‐D differential gel electrophoresis found 49 proteins differentially regulated in the SFG and 94 proteins in the OC. Upon comparison of these two cortical regions, 23 overlapping proteins were identified. Further analysis of these overlapping proteins using mass spectrometry revealed a common role in synaptic activity (Etheridge et al. 2009). Furthermore, antibody microarray focused on the catenin signaling pathway in the SFG found a significant increase in α‐ and β‐catenin levels, indicative of neuroadaptive changes (Al‐Housseini et al. 2008) Immunoblotting in the human post‐mortem PFC revealed increased synaptophysin I in subjects with AUD (Henriksson et al. 2008). Synaptophysin I modulates synaptic strength and is thought to localize at glutamatergic synapses; therefore, the increase in levels of this protein in AUD indicates there are anomalies of glutamate circuitry in the PFC (Henriksson et al. 2008).

Overall, the changes in the cortex are either cortical degeneration, a causal factor of the weakening of cognitive control, or reorganisation of neural networks driven by the vast number of cytoskeletal and synaptic proteins altered. These may contribute to the physical remodelling of neuronal circuits, altering their architecture and enhancing susceptibility to addictive behaviors.

### 2.4 Potential Clinical Targets

Oxytocin, which has been proposed as an anti‐craving medication, reverses the social behavior impacted by long‐term drug use (McGregor and Bowen 2012). Oxytocin receptor binding was increased in AUD compared to controls in the dlPFC BA9 (and the nucleus caudate and ventral striatum discussed below), and increased oxytocin receptor mRNA levels in AUD were measured using qRT‐PCR. In the same study, fMRI analysis on 12 heavy social drinkers with intranasal oxytocin administration reduced reactivity to alcohol‐related cues in the frontal lobe (Hansson et al. 2018). The effect of intranasal oxytocin in AUD suggests that oxytocin levels are lower while oxytocin receptor expression is increased. This may be due to the loss of oxytocin‐producing neurons (paraventricular nucleus) in AUD (Hansson et al. 2018). Notably, oxytocin can act independently of oxytocin receptors, particularly under ethanol‐induced conditions where oxytocin can block GABA actions at δ subunit‐containing GABAA receptors (δ‐GABAARs) (Bowen et al. 2015). Overall, these studies in the PFC have unveiled crucial insights into the molecular underpinnings of alterations caused by AUD. This includes reductions in white matter, neurons, and metabolic enzymes, with insights into apoptotic pathways and neurotransmitter changes.

## 3 Striatum

The striatum plays a significant role in AUD as it is implicated in the reward system and addiction‐related processes, such as reward, reinforcement, learning, and habit formation (Blaine et al. 2020). Specifically, the ventral striatum (or nucleus accumbens) and caudate mediate neuroplasticity associated with the development and maintenance of addictive behaviors (Balleine et al. 2007; Floresco 2015). A recent meta‐analysis study conducted across species on the prefrontal cortex, nucleus accumbens, and amygdala from AUD humans and animal models reported differentially expressed genes in the PFC and amygdala but not in the nucleus accumbens, suggesting the nucleus accumbens plays a role in the development of alcohol‐seeking behavior but not in the maintenance of addiction behavior (Friske et al. 2025). However, comparison between the ventral and dorsal striatum between individuals with AUD and control cases revealed differentially expressed genes only in the dorsal striatum (Zillich et al. 2022). Such molecular studies demonstrate subregional differences within the striatum in AUD, which suggests a broader network reorganization in which striatal connectivity with cortical and limbic circuits leads to loss of flexibility and strengthening of pathological habit circuits. Such network shifts help explain the persistence of compulsive drinking despite negative consequences.

Expression of μ‐opioid receptor (MOR) and its activity have also been examined in the ventral striatum and caudate of AUD subjects due to the known effects of alcohol on the endogenous opioid system (Hermann et al. 2017). Convergent data from both post‐mortem and clinical PET analysis of the striatum provided insight into the dynamics of the endogenous opioid system as well as MOR activity in AUD. Using qRT‐PCR and receptor autoradiography, mRNA and MOR‐binding sites were reduced in the striatum of AUD cases. This finding aligned with data from clinical PET analysis, which found a lower binding potential for [^11^C] carfentanil (radiolabelled opioid analgesic), which was associated with an increased risk for relapse (Hermann et al. 2017). Oxytocin receptor expression and binding have also been examined using qRT‐PCR and receptor autoradiography in the nucleus caudate and ventral striatum, where there was a significant increase in oxytocin receptor binding sites in AUD (Hansson et al. 2018). These findings in human post‐mortem brains validated findings from concurrent rodent studies, which also reported increased oxytocin mRNA and binding in the striatum in the AUD rodent model (Hansson et al. 2018). Genome‐wide RNA sequencing in the caudate‐putamen of human AUD identified 171 upregulated and 132 downregulated genes in individuals with AUD compared with controls. Upon closer analysis, the authors found significant downregulation of M4 muscarinic acetylcholine receptor (mAChR) at the gene and protein levels in the putamen, but not in the caudate of AUD cases (Walker et al. 2020). A rodent model of AUD reported reduced expression of M4 mAChR in the dorsolateral striatum, which is specific to dopamine D1 receptor‐expressing medium spiny neurons. Systemic administration and local microinjections of the selective M4 mAChR positive allosteric modulator (VU0467154) reduced alcohol seeking (in home cage access, cue induced reinstatement, and self‐administration) (Walker et al. 2020). Together, the human post‐mortem and rodent analysis suggest that the reduction of M4 mAChR in the striatum may facilitate alcohol seeking, and activation of these receptors, potentially on D1 receptor‐expressing medium spiny neurons, may play an important role in reducing alcohol‐seeking behaviors. Striatal subregional analysis of the putamen, globus pallidus, and ventral pallidum showed microglia, but not astrocytes, underwent significant morphological changes due to prolonged alcohol exposure. The activation or dystrophic microglia phenotype observed in AUD cases was characterized by decreased cell size and process retraction. Importantly, these microglial changes were reversed in individuals who had remission from AUD for at least 1 year, suggesting that abstinence can restore microglia function in striatal regions (Rasool et al. 2024). Based on the functions of the striatum in reward processing, changes reported within the striatum in AUD cases are linked to alcohol seeking behavior, making this region a compelling target for preventing relapse through opioid, oxytocin, mAChR, or microglial manipulation (Lee et al. 2024; Walker et al. 2024).

## 4 Hippocampus

The hippocampus is a brain region that encodes spatial memory and is sensitive to the effects of alcohol use; for instance, clinical neuroimaging studies in patients with AUD show reduced volume of the hippocampus (Cornish and Prasad 2021; De Bellis et al. 2000; Geuze et al. 2005). Post‐mortem studies report that the reduction in hippocampal volume in AUD is mainly due to reduced white matter with no change in the number of neurons within hippocampal subregions (CA1, CA2, CA3, CA4, subiculum, presubiculum, and dentate gyrus). Notably, this study included alcoholic Korsakoff's psychosis, where the finding was similar to chronic alcoholics (Harding et al. 1997). The lack of change in neuronal loss was supported by another independent study that examined the hippocampal subregions of the granule, hilus, CA1/CA2/CA3, and subiculum. However, glial cells were most significantly reduced globally in the hippocampus in AUD compared with controls. Astrocytes and oligodendrocytes were more impacted than microglia in AUD cases (Ikegami et al. 2003; Korbo 1999; Liu et al. 2021).

To establish the mechanism behind these functional and structural changes seen in the hippocampus of individuals with AUD, protein profiles have been analyzed using 2D electrophoresis in human post‐mortem tissue (Matsuda‐Matsumoto et al. 2007). The proteins that were altered in the hippocampus in AUD can be classified into two functional groups responsible for metabolism (oxidative stress, vesicle trafficking) and cytoskeletal characteristics, where a decrease in expression was mostly found (Matsuda‐Matsumoto et al. 2007). Moreover, through comparisons with protein profiles of other brain regions, such as BA9 white matter, it can be seen that there are differential protein expression profiles across brain regions; for instance, transketolase (a thiamine‐related protein) was altered in BA9 white matter but not in the hippocampus (Matsuda‐Matsumoto et al. 2007). A study that analyzed glucagon‐like peptide‐1 (GLP‐1) expression in the amygdala, ventral tegmental area, nucleus accumbens, hippocampus, and prefrontal cortex multi‐brain found a significant fold change of GLP‐1R mRNA only in the hippocampus AUD compared to controls (Farokhnia et al. 2022). In addition to comparisons across brain regions, subregional analysis is also important. For example, using immunohistochemistry, an increase in the expression of hNP22, a cytoskeletal protein found on neurons, was shown in the CA3 and CA4 hippocampal subregions (Depaz et al. 2003), strengthening the idea that alcohol may have regionally selective effects on neurons. More recently, changes in microRNA (miRNAs) have also been identified. MicroRNA‐34a and MicroRNA‐34c were upregulated in the hippocampus in AUD cases (Santos‐Bezerra et al. 2021; Wang et al. 2024). MicroRNAs may participate in the pathogenesis of AUD by modulating downstream target hippocampal genes. Together, these changes may impact hippocampal contributions to memory and contextual processing, increasing vulnerability to memory associations to relapse to alcohol seeking (Meda et al. 2018).

## 5 Ventral Tegmental Area and Substantia Nigra

The ventral tegmental area (VTA) is central to reward pathways in the reinforcing effects of alcohol and the development of addiction (Kalivas and Volkow 2005). Human post‐mortem studies on the VTA are limited; however, measurement of a potent GABAergic steroid that modulates the activity of GABA_A_ receptors, 3‐hydoxy‐pregnane‐20‐one (3α, 5α‐THP), has shown interesting results. Using immunohistochemistry, the expression of 3α, 5α‐THP was increased in the VTA of AUD subjects compared to controls (Hasirci et al. 2017). Interestingly, in the same study, analysis in the substantia nigra pars medialis (SNM) overall showed no difference between groups. However, sex‐specific effects of AUD were found, wherein levels of 3α, 5α‐THP increased in male AUD subjects compared to male controls only (Hasirci et al. 2017). In rodent AUD studies, alcohol consumption has also shown increased 3α, 5α‐THP in male mice (Finn et al. 2004). Given the mechanism of 3α, 5α‐THP to modulate GABA_A_ receptors, this steroid may be important for the behavioral effects of short‐ and long‐term alcohol use (Hasirci et al. 2017). Moreover, 3α, 5α‐THP was found to be primarily localized in dopaminergic neurons using double fluorescence labeling (Hasirci et al. 2017), also implicating a possible role for this steroid in regulating AUD. Changes in neuroinflammation were assessed by measuring concentrations of MCP‐1 in multiple brain regions using an ELISA‐based assay and immunohistochemistry for Glut5, a microglia marker (He and Crews 2008). MCP‐1 is a cytokine that facilitates the migration and activation of microglia. Increased levels of MCP‐1 and Glut5 were found in the VTA, SN, and amygdala, which could lead to increased levels of proinflammatory, neurotoxic cytokines, thereby providing a potential explanation for the mechanism behind neurodegeneration in the VTA and SN in AUD (He and Crews 2008). Increased inflammation has been shown to alter dopamine transmission in mesolimbic and mesocortical pathways, resulting in network transitions that shift motivation toward dysfunctional states associated with addiction (Felger and Treadway 2017).

## 6 Corpus Callosum

The corpus callosum is composed of white matter of fibre tracts and facilitates communication and coordination between the left and right hemispheres (Mooshagian 2008). Volumetric examination of the corpus callosum has revealed a reduction in volume in AUD subjects, with shrinkage in the genu and splenium being the most prominent (Pfefferbaum et al. 2006; Tarnowska‐Dziduszko et al. 1995). Moreover, diffusion tensor imaging studies show that voluntary chronic alcohol consumption alters the microstructural integrity of the corpus callosum, which by extension accompanies macrostructural shrinkage (Pfefferbaum et al. 2006) Using 2D electrophoresis and mass spectrometry, it was found that in the splenium and genu of the corpus callosum, the process of lipid peroxidation is altered due to increased expression of phospholipase D in AUD cases (Kashem et al. 2008; Kashem et al. 2007). In the presence of ethanol, phospholipase D produces phosphatidylethanol (PEth). PEth is a phospholipid that increases membrane fluidity, where changes in PEth levels can lead to cellular damage. However, no change in phospholipase D was found in the BA9 of white matter in AUD human post‐mortem tissue, thereby suggesting that there are specific changes to this protein expression and that different regions of white matter may be differently impacted by chronic alcohol use (Kashem et al. 2008).

## 7 Hypothalamus

The hypothalamus modulates feeding and social behavior, including alcohol consumption (Barson and Leibowitz 2016). Immunohistochemistry analysis revealed the vulnerability of the hypothalamus to neuronal degeneration in AUD, where neuronal loss primarily affected the supraoptic nucleus (SON), with additional neuronal loss observed in the paraventricular nucleus (PVN) in cases with extended alcohol consumption histories, irrespective of other common medical complications in AUD (Harding et al. 1996). While significant neuronal loss occurred in the SON of chronic alcohol use in humans, there was also a notable reduction in the overall size of the SON and a decrease in vasopressin‐expressing neurons (Harding et al. 1996). Both vasopressin and oxytocin are produced in the PVN and SON of the hypothalamus (Møller 2021). Hence, neuronal loss in the SON and PVN found in AUD would directly reduce vasopressin and oxytocin levels, key neuromodulators that regulate social behavior. Degeneration of the PVN and SON in AUD may be the causal effect of decreased oxytocin receptor expression in the PFC, indicating how local neuronal loss in one region may impact brain network function in social and reward networks (Hansson et al. 2018).

## 8 Cerebellum

The cerebellum plays a role in modulating and refining motor skills and balance, as well as cognitive processes controlling movement, which may be impaired following chronic alcohol use (Nuñez‐delMoral et al. 2023; Sullivan et al. 1995). The cerebellum is particularly vulnerable in individuals with alcoholism, as chronic alcohol abuse often leads to thiamine deficiency, a key factor in Wernicke–Korsakoff syndrome (Harper and Kril 1990). Within the cerebellum, the vermis is most impacted in alcohol‐related diseases (de la Monte and Kril 2014). Examination of neuronal numbers within the cerebellum revealed decreased flocculi and vermal Purkinje cell density, and white matter atrophy within thiamine‐deficient AUD subjects (Baker et al. 1999). Disruption to Purkinje cells in the cerebellum can, by extension, also disrupt the spinocerebellar and vestibulocerebellar pathways, affecting normal cerebellar functioning in AUD (Baker et al. 1999). Analysis of five cerebellar subregions (the anterior and posterior lobes, the anterior and posterior vermi, and the flocculonodular lobe) showed no significant loss. However, analysis of the cerebellum overall revealed the average volume of Purkinje cell perikaryon was reduced by ~20% along with a decrease in the Purkinje cell nuclei by 16% (Andersen 2004; Phillips et al. 1987). This degeneration of Purkinje cells weakens the cerebellar outputs to both motor and associative cortical regions, potentially contributing to the shift from goal‐directed to habitual behavior in AUD.

## 9 Future Directions

In summary, most brain regions investigated in post‐mortem AUD tissue show some level of degeneration and molecular dysregulation that extends beyond local pathology to influence network level connectivity. Neurodegeneration in the PFC affects white matter integrity, hippocampal glial loss undermines memory circuits, hypothalamic neuropeptide loss disrupts stress and reward loops, and cerebellar degeneration impairs cognitive to motor integration. Interestingly, no degeneration has been reported in the striatum. The number of parvalbumin or calretinin neurons changed in the ventral pallidum in AUD compared to controls, suggesting molecular changes within the basal ganglia may cause functional changes promoting alcohol seeking (Rasool et al. 2024). Across all brain regions discussed here, there are several proteomic changes; the key proteins altered in specific brain regions in AUD are summarised in Table 1.

**TABLE 1:** Summary of cellular alterations across brain regions in Alcohol Use Disorder (AUD).

Brain region	Pathology
Prefrontal Cortex	‐Reduced volume of grey and white matter in the PFC in AUD ‐Decrease in the white matter of prefrontal, middle temporal, and visual cortices ‐Decrease in neurons in the frontal cortex *Structural proteins* ‐Reduction in selective lipids; sphingolipids (sulfatides and ceramides), and phospholipids ‐Decrease in cytoplasmic protein (hNP22), cytoskeletal protein (α‐Internexin) ‐Increased level of synaptophysin I ‐Decrease in metabolic enzymes (Creatine Kinase chain B, fructose‐biphosphate aldolase C, and glyceraldehyde‐3‐phosphate dehydrogenase) *Apoptosis‐related* ‐Increase in pro‐apoptotic protein, caspase‐3, ‐Increase anti‐apoptotic protein, B‐cell lymphoma‐2 ‐In the orbitofrontal cortex, increase of toll‐like receptor 7 (TLR7) and tumour necrosis factor‐related apoptosis ‐Upregulation of endoplasmic reticulum stress‐associated proteins, including glucose‐regulated protein 78, oxidative stress markers ‐Increase interferon gamma (IFNγ), protein kinase R, and phosphorylated PKR
Striatum	No change in astrocytes Change in microglia morphology Reduction in μ opioid receptor mRNA and binding sites Increase in oxytocin receptor binding sites A decrease in M4 muscarinic acetylcholine receptor in the putamen
Hippocampus	Reduction in hippocampal volume No change in neurons Decreases in glia Increase GLP‐1R mRNA Increase in MicroRNA‐34a and MicroRNA‐34c
VTA/SN	Increase in 3α, 5α‐THP, MCP‐1 and Glut5
Corpus callosum	Reduced in volume in AUD subjects, with shrinkage in the genu and splenium Increase phospholipase D
Hypothalamus	Neuronal loss primarily in supraoptic nucleus and paraventricular nucleus (PVN)
Cerebellum	Reduction in cerebellum volume Loss of Purkinje cell perikaryon

Studies using AUD human post‐mortem brain tissues reviewed here have collectively advanced our understanding of the impact of alcohol not only in neuropathology but also in the molecular alterations that impact the complexity of AUD. Further, expansion of how these local cellular changes lead to network reorganization could be achieved using current neuroscience tools, such as spatial transcriptomics and proteomic tools. Reflecting on the current advancement in neuroscience, including the approaches below, future AUD research will greatly enhance outcomes from AUD human post‐mortem brain tissues. Table 2 summarizes the current challenges and potential strategic approaches.

**TABLE 2:** Current challenges and strategies in studying Alcohol Use Disorder (AUD).

Challenges	Potential strategy
Examination of multiple brain regions	‐Inclusion of multiple brain regions ‐Analysis of subregional differences
Gender effects	‐Report the number of females in studies ‐Inclusion of sex analysis ‐Ensure to include females in studies
Integration of data from animal models for translational impact	‐Identify common or discoveries cellular changes reported in rodent and monkey AUD studies to be examined in human studies using Meta analysis approach ‐Examine cellular changes identified from pre‐clinical studies
Application of new technologies	‐Spatial transcriptomics ‐Spatial Mass Spectrometry ‐Mass Spectrometry (Lipidomics, proteomics)

### 9.1 Multiple Brain Regional Analysis

Comprehensive analysis of several brain regions together would allow the identification of selective and common changes in AUD. Analysis of GLP‐1 mRNA in the amygdala, VTA, NAc, hippocampus, and PFC revealed an increase of GLP‐1 only in the hippocampus and PFC. Analysis of multiple regions provides mechanistic insight into GLP‐1 actions in AUD (Farokhnia et al. 2022). Another example is Hevin, a matricellular matrix protein found in astrocytes, parvalbumin interneurons, and glutamatergic neurons, which promotes the formation of excitatory synapses and plays a role in the maintenance of dendritic spines (Nuñez‐delMoral et al. 2023). Using western blot and RT‐qPCR approaches in multiple brain regions showed increased levels of Hevin in PFC, striatum, amygdala, hippocampus, and cerebellum (Nuñez‐delMoral et al. 2023), and indicates that neuroplastic changes are common across these regions in AUD. The increase in Hevin identified across multiple brain regions supports neuroplastic changes and highlights a common change in neuroplasticity in AUD (Nuñez‐delMoral et al. 2023). Using liquid chromatography–tandem mass spectrometry (LC–MS/MS) analysis of human postmortem tissue, six brain regions were analysed (amygdala, hippocampus, hypothalamus, nucleus accumbens, prefrontal cortex, and ventral tegmental area); the amygdala had the highest differential expression of proteins (Teng et al. 2023). Although the cortex, striatum, and hippocampus are most studied in AUD, this study found that the amygdala showed the greatest number of differentially expressed proteins. Analysis of multiple brain regions can advance the field by understanding the shared mechanism of degeneration or factors that contribute to compulsive alcohol seeking behaviours.

### 9.2 Incorporation of Sex‐Specific Analyses

Alcohol is the most prominent substance of abuse among females (Verplaetse et al. 2021), and as such, the incorporation of sex analysis should be included in AUD studies (Cornish and Prasad 2021). Especially with epidemiological studies revealing a significant increase in AUD in females compared to males (Grant et al. 2017). A large‐scale neuroimaging study compared amygdala and hippocampus volumes in AUD and controls and conducted sex difference analysis. This study found males with AUD had smaller volumes of the total amygdala and hippocampus regions compared to male controls. In contrast, the CA1 and subiculum subfield volumes were reduced in both males and females with AUD when compared to healthy sex‐matched controls (Grace et al. 2021). Based on imaging studies, the interaction of AUD with sex suggests significant cellular changes in post‐mortem human brain in AUD cases. However, limited studies have incorporated sex analysis. A great example of the implications of sex inclusion and analysis was reported by Hansson and Spanagel (2021), where oxytocin levels decreased and oxytocin receptor binding sites increased in male AUD, yet no change in the oxytocin system was detected in female AUD cases (Hansson and Spanagel 2021). This study showcases the importance of sex analysis for clinical understanding and interventions for AUD.

### 9.3 Incorporation of Findings From Animal Models in Human Post‐Mortem

Potential therapeutic mechanisms have been identified (oxytocin and GLP‐1) through the integration of basic research from rodent models of AUD, emphasizing the value of basic science in understanding and treating AUD. Findings from animal models of addiction have highlighted several underexplored brain regions in human AUD, including the anterior insular cortex, nucleus accumbens, ventral pallidum, and bed nucleus of the stria terminalis (Campbell et al. 2019; Gibson et al. 2018; Pleil et al. 2024; Prasad and McNally 2016, 2020; Prasad and Wallén‐Mackenzie 2024). These preclinical insights can inform the investigation of these regions in human post‐mortem tissue. Moreover, emerging studies are beginning to incorporate new technologies with co‐designing of cross‐species analysis and including multiple brain regions (Friske et al. 2025; Willis et al. 2025; Zillich et al. 2025). A meta‐analysis study identified a combination of differentially expressed genes across three brain regions common in rodent, monkey, and human AUD cases. Incorporating findings from animal models can aid in the identification of not only biomarkers for AUD but also feasible therapeutic targets.

## 10 Conclusions

AUD is a complex neurological disorder and requires a multidisciplinary approach to improve our understanding of the neurobiological underpinnings for treatment discovery. The investigation of postmortem brain tissue from subjects with AUD is a fundamental resource and has provided valuable insights into the molecular mechanisms and structural changes associated with AUD. Overall, findings presented in this review illustrate that AUD neuropathology is not confined to isolated regions but reflects a broader reorganization of interconnected neural networks. Understanding how cellular and molecular changes rewire cortical, striatal, limbic, and cerebellar circuits provides a critical framework for developing targeted interventions.

Together with pre‐clinical, human neuroimaging studies and the incorporation of emerging neuroscience tools, the integration of post‐mortem findings is a powerful approach and forms a crucial bridge for understanding the complexity of AUD.

## Author Contributions

**Ameer E. Rasool:** writing – original draft. **Jennifer L. Cornish:** supervision, writing – review and editing, funding acquisition. **Asheeta A. Prasad:** conceptualization, writing – original draft, supervision, writing – review and editing, visualization, project administration, funding acquisition.

## Conflicts of Interest

The authors declare no conflicts of interest.

## Peer Review

The peer review history for this article is available at https://www.webofscience.com/api/gateway/wos/peer‐review/10.1111/jnc.70233.

## Data Availability

Data sharing not applicable to this article as no datasets were generated or analysed during the current study.
